# Laboratory test availability and user fees in routine HIV care: a global analysis in 42 countries

**DOI:** 10.1093/haschl/qxag151

**Published:** 2026-06-13

**Authors:** Zhongzhe Pan, Ellen Brazier, Stephany N Duda, Jeremy Ross, David J Templeton, Sanjay Pujari, Lauren C Zalla, Ronald M Galiwango, Geoffrey Fatti, Gad Murenzi, Gabriela Carriquiry, Henri Chenal, Romanee Chaiwarith, Raynell Lang, Helen Byakwaga, Chido Chinogurei, Peter Ebasone, Carina Cesar, Oliver Ezechi, Aimee Freeman, Lameck O Diero, Nicola van Dongen, Patricia Lelo, Sandra Wagner Cordoso, Ephrem Mensah, Kathleen McGinnis, Francesca Odhiambo, Ethel Rambiki, Christella Twizere, Marco Luque, Armel Poda, April D Kimmel

**Affiliations:** Department of Health Policy, Virginia Commonwealth University School of Public Health, Richmond 23219, United States; Institute for Implementation Science in Population Health, City University of NewYork, Graduate School of Public Health and Health Policy, New York 10027, United States; Department of Biomedical Informatics, Vanderbilt University Medical Center, Nashville 37203, United States; TREAT Asia, amfAR—the Foundation for AIDS Research, Bangkok 10110, Thailand; Department of Sexual Health Medicine, Sydney Local Health District and Faculty of Medicine and Health, University of Sydney, Sydney 2006, Australia; Institute of Infectious Diseases, Pune 411037, India; Department of Epidemiology, Johns Hopkins Bloomberg School of Public Health, Baltimore 21205, United States; Rakai Health Sciences Program, Kyotera, Uganda; Division of Epidemiology and Biostatistics, Department of Global Health, Faculty of Medicine and Health Sciences, Stellenbosch University, Cape Town 7599, South Africa; School of Public Health, College of Medicine and Health Sciences, University of Rwanda, Kigali, Rwanda; Instituto de Medicina Tropical Alexander von Humboldt, Universidad Peruana Cayetano Heredia, Lima 31, Peru; Centre Intégré de Recherches Biocliniques d'Abidjan, Abidjan 18, Côte d'Ivoire; Faculty of Medicine and Research Institute for Health Sciences, Chiang Mai University, Chiang Mai 50200, Thailand; Department of Medicine, University of Calgary, Calgary, Alberta, Canada T2N 1N4; Faculty of Health Sciences, Mbarara University of Science and Technology, Mbarara, Uganda; Centre for Integrated Data and Epidemiological Research, School of Public Health, University of Cape Town, Cape Town 7925, South Africa; Department of Research, Clinical Research Education, Networking and Consultancy (CRENC), Yaoundé, Cameroon; Fundacion Huesped, Buenos Aires C1427, Argentina; Centre for Reproductive and Population Health Studies, Nigerian Institute of Medical Research, Lagos 101245, Nigeria; Department of Epidemiology, Johns Hopkins Bloomberg School of Public Health, Baltimore 21205, United States; Department of Medicine, Moi University, Eldoret 30100, Kenya; Empilweni Services and Research Unit, Department of Paediatrics and Child Health, Rahima Moosa Mother and Child Hospital, University of the Witwatersrand, Johannesburg 2000, South Africa; Unit of Infectious Diseases, Kalembelembe Pediatric Hospital, Kinshasa 00000, Democratic Republic of Congo; Fiocruz-Instituto Nacional de Infectologia (INI), Rio de Janeiro 21040-360, Brazil; Espoir Vie Togo, Lomé, Togo; Department of Internal Medicine, VA Connecticut Healthcare, West Haven 06516, United States; Family AIDS Care and Education Services, Kenya Medical Research Institute, Kisumu 40100, Kenya; Lighthouse Clinic Trust, Lilongwe 207233, Malawi; Centre National de Référence en Matière de VIH/SIDA, Bujumbura, Burundi; Instituto Hondureño de Seguridad Social, Tegucigalpa 11101, Honduras; Department of Infectious Diseases, Sourô Sanou Teaching Hospital, Bobo-Dioulasso, Burkina Faso; Department of Health Policy, Virginia Commonwealth University School of Public Health, Richmond 23219, United States; Division of Infectious Diseases, Department of Internal Medicine, Virginia Commonwealth University School of Medicine, Richmond 23298, United States

**Keywords:** HIV, laboratory testing, availability, user fees, global health, donor support

## Abstract

**Introduction:**

Laboratory testing is critical for diagnosis and treatment management for people with HIV, but it is costly. When HIV resources are constrained, even before recent disruptions in US funding for the global HIV response, user fees have been used for cost recovery. We describe the availability and charging of user fees for routine HIV laboratory testing globally.

**Methods:**

Routine test availability and charging of user fees were examined via an HIV clinic survey conducted September 2020–March 2021; responses reflected clinical practices in 2019. HIV clinics came from 42 low-, middle-, and high-income countries from the International epidemiology Databases to Evaluate AIDS (IeDEA) global research consortium.

**Results:**

Across 199 clinics, on-site test availability was generally limited, reflecting constraints in routine test availability across health conditions more broadly. Over 90% of clinics did not report charging user fees. A higher percentage of reporting user fees was from middle-income countries and from countries receiving Global Fund or PEPFAR country support, which may reflect settings with ongoing health funding gaps.

**Conclusion:**

Availability and user fees for essential HIV-related laboratory tests were limited in 2019. Monitoring user fees may provide insights about how national health systems are responding to steep reductions in global donor funding.

Key points

- On-site availability of laboratory tests for routine HIV diagnosis and management was limited in a diverse global cohort of HIV clinics in 2019.
- Most clinics in this cohort did not report user fees, although this varied by country income status and sources of donor funding for HIV.
- Monitoring user fees for essential HIV-related laboratory tests may provide important insights about how national health systems are responding to steep reductions in global HIV and other donor funding.

## Introduction

Laboratory testing is important for diagnosis and treatment management for people with HIV,^1^ but it can be costly.^2^ Higher laboratory test costs, particularly for specialized tests such as genotypic resistance testing, can limit test availability and critical access to HIV diagnosis, treatment, and care.^3^ Even if tests are available, higher costs may prompt cost-recovery efforts like user fees, ie, fixed payments imposed by facilities on patients for healthcare, medication, or supplies at the point-of-service.^4,5^ User fees disproportionately affect those with greater medical needs and increase disparities in healthcare access.^6-10^ HIV-related user fees were largely eliminated in the early 2000s,^6^ with ongoing discouragement by global health stakeholders.^6,11-14^ However, resource constraints in some settings have led to their reintroduction.^15-18^

Little is known about the recent landscape of HIV-related laboratory test availability and user fees globally. We describe availability and charging of user fees for routine HIV-related diagnosis and laboratory testing among HIV treatment and care clinics participating in the International epidemiology Databases to Evaluate AIDS (IeDEA) global research consortium, contextualizing practices by site- and country-level characteristics.

## Methods

### Data

Data on laboratory testing availability and charging of user fees, although not fee waivers or other exemption policies, came from a survey distributed to 238 IeDEA-participating clinics in 2020, including clinics in 42 countries in the Asia-Pacific; the Caribbean, Central America, and South America; North America; and Central, East, Southern, and West Africa. The survey explored site-level characteristics and HIV-related services during 2019, with responses reflecting routine clinic services and practices before the SARS-CoV-2 pandemic.^19^ Designated non-human subjects research by Vanderbilt University Medical Center's IRB (#200013), the survey was conducted between September 2020 and March 2021^20,21^ and had a response rate of 95%.^19^

Data also included country-level characteristics for 2019. World Bank country-level income level was low, low-middle, middle-high, or high.^22^ Country-level US President's Emergency Plan for AIDS Relief (PEPFAR) support was any country with a PEPFAR country operational plan^23^ and Global Fund to Fight AIDS, Tuberculosis and Malaria (Global Fund) support was any country receiving Global Fund allocations.^24^ National HIV prevalence (≤1%, >1%-5%, >5%) came from UNAIDS.^25^

### Sample

The analytic sample included HIV clinics responding to the survey and serving adults. We excluded clinics serving children only, given clinical practice differences for pediatric patients.

### Survey measures

The survey examined on-site availability (ie, provided in the HIV clinic or within the same health facility) of routine laboratory tests. Tests included: HIV-1 p24 antigen test for acute HIV-1 infection (p24), HIV-1/HIV-2 antigen/antibody immunoassay for established HIV infection (HIV-1/2 Ag/Ab), supplemental HIV-1/HIV-2 antibody differentiation immunoassay (HIV-1/2 Ab suppl), CD4 cell count (CD4),^26^ quantitative PCR for HIV viral load, and HIV-1 genotypic drug resistance testing (genotyping).

User fees were any fees, including partial contributions, other than insurance co-pays, paid by service users for a test. Insurance co-pays were not considered, given our focus on user fees as a facility-level cost-recovery mechanism. Tests were classified as having, not having, or unknown user fees.

### Analysis

We report frequencies and percentages of test availability and user fees for each test, conditional on their availability, overall and by region, clinic population urbanicity, and country-level characteristics. In a subgroup analysis of clinics in low-and lower-middle-income countries (all funded by the Global Fund), we examined differences between PEPFAR-supported and non-PEPFAR countries. Analyses were performed using RStudio (version 2026.01.0, Posit Software, PBC).

## Results

### Clinic characteristics

Of 238 clinics surveyed, 199 (84%) responded to the survey and provided adult HIV care (Table 1). Most were in East Africa (37%), served urban populations (64%), were in countries receiving Global Fund (72%) and PEPFAR (69%) support, and were lower-middle-income (37%).

**Table 1:** Summary of clinic characteristics.

Characteristic	Clinics, *N* (%)^a^
Overall	199 (100)
IeDEA region	
Asia-Pacific	37 (19)
Central Africa	20 (10)
CCASAnet	7 (4)
East Africa	74 (37)
NA-ACCORD	30 (15)
Southern Africa	25 (13)
West Africa	6 (3)
Clinic population urbanicity	
Urban	127 (64)
Rural	68 (34)
Country income group	
Low	49 (25)
Lower-middle	74 (37)
Upper-middle	24 (12)
High	52 (26)
Country Global Fund support	
Supported	143 (72)
Not supported	56 (28)
Country PEPFAR support	
Supported	137 (69)
Not supported	62 (31)
Country HIV prevalence	
≤1%	67 (34)
>1%-5%	66 (33)
>5%-100%	57 (29)

Abbreviations: IeDEA, International epidemiology Databases to Evaluate AIDS; avail, available; CCASAnet, Caribbean, Central and South America network for HIV Epidemiology; NA-ACCORD, North American AIDS Cohort Collaboration on Research and Design; Global Fund, Global Fund to Fight AIDS, Tuberculosis and Malaria; PEPFAR, U.S. President's Emergency Plan for AIDS Relief.

^a^The percentages reported are column percentages of all clinics. Column percentages may not sum to one due to rounding and because we do not report clinics with missing information.

### Availability

**Figure 1.**
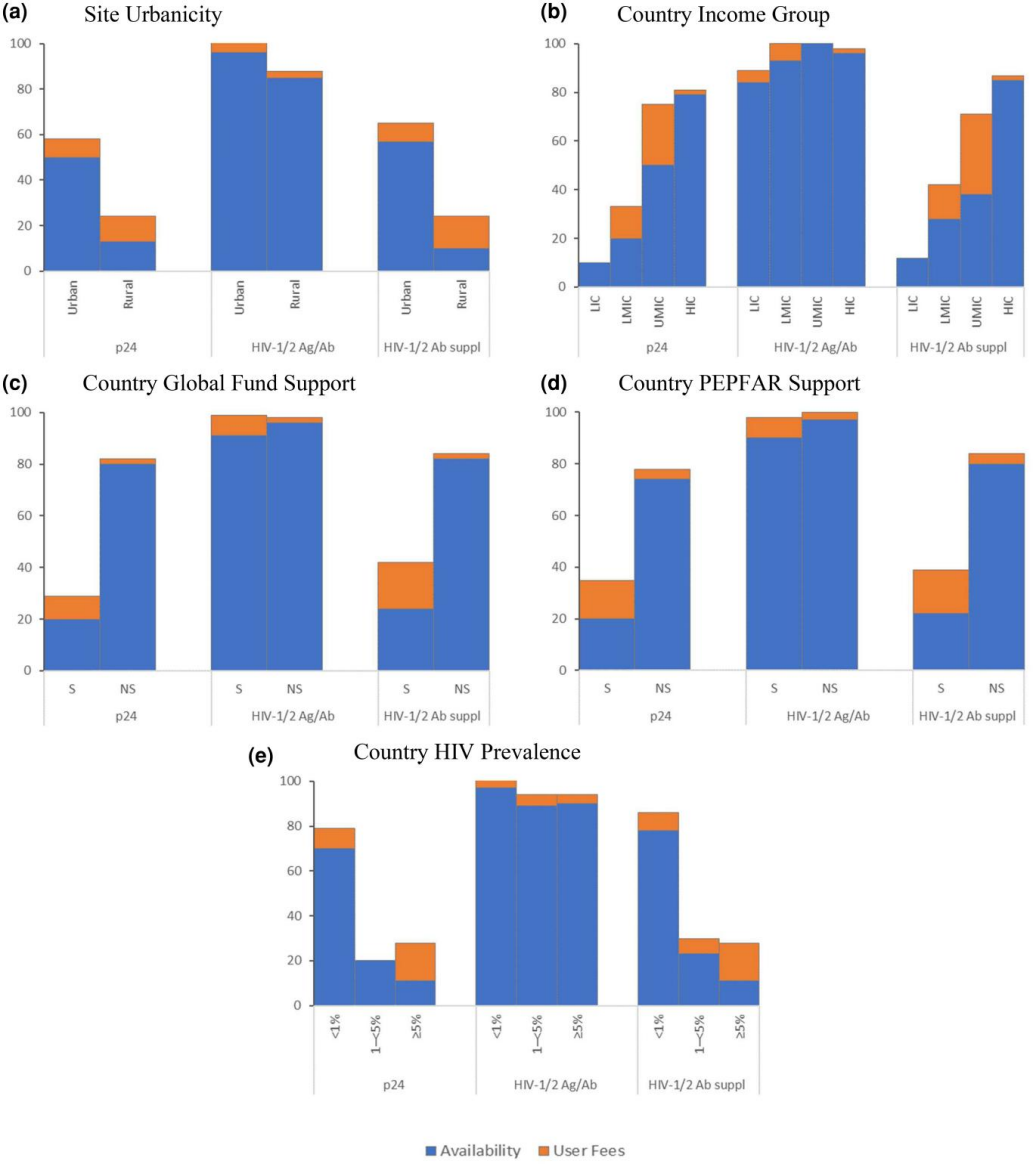
Routine laboratory tests for HIV diagnosis: availability and user fees, %. Abbreviations: p24, HIV-1 p24 antigen test for acute HIV-1 infection; HIV-1/2 Ag/Ab, HIV-1/HIV-2 antigen/antibody immunoassay test for established HIV infection; HIV-1/2 Ab suppl, HIV-1/HIV-2 antibody differentiation immunoassay; LIC, low-income country; LMIC, lower-middle-income country; UMIC, upper-middle-income country; HIC, high-income country; Global Fund, Global Fund to Fight AIDS, Tuberculosis and Malaria; S, Supported; NS, Not supported; PEPFAR, President's Emergency Plan for AIDS Relief. Shown are the percentage of clinics with available laboratory tests for HIV diagnosis and the percentage of clinics reporting user fees conditional on the availability of a given diagnostic test. Availability was defined as on-site availability (ie, provided in the HIV clinic or within the same health facility). User fees were defined as fees other than insurance co-pays paid by service users for a given laboratory test.

**Figure 2.**
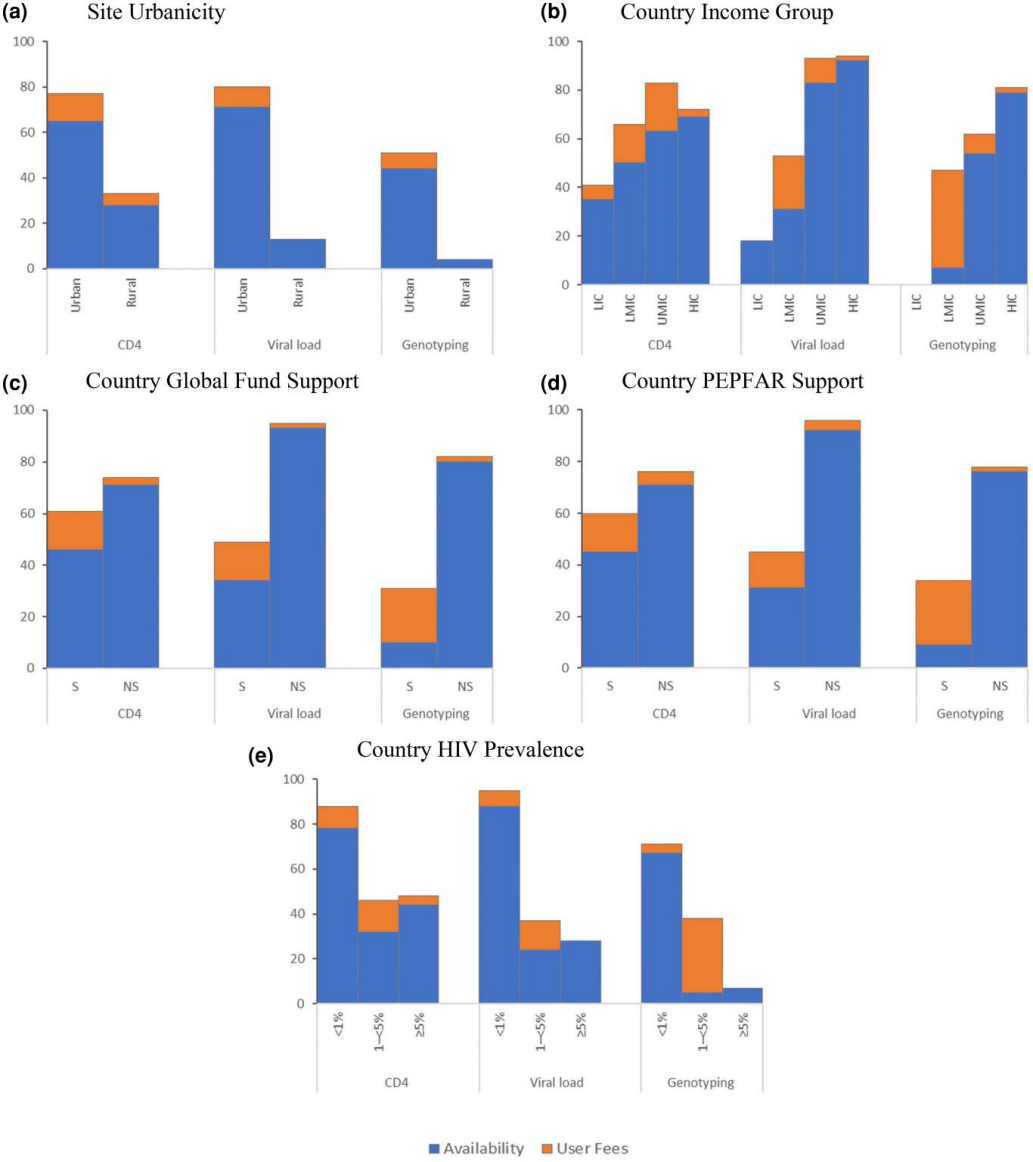
Routine laboratory tests for HIV management: availability and user fees, %. Abbreviations: CD4, CD4 cell count test; viral load, quantitative PCR for HIV viral load; genotyping, HIV-1 genotypic drug resistance testing; LIC, low-income country; LMIC, lower-middle-income country; UMIC, upper-middle-income country; HIC, high-income country; Global Fund, Global Fund to Fight AIDS, Tuberculosis and Malaria; S, supported; NS, not supported; PEPFAR, President's Emergency Plan for AIDS Relief. Shown are the percentage of clinics with available laboratory tests for HIV management and the percentage of clinics reporting user fees conditional on the availability of a given test. Availability was defined as on-site availability (ie, provided in the HIV clinic or within the same health facility). User fees were defined as fees other than insurance co-pays paid by service users for a given laboratory test.

On-site test availability varied widely (Figures 1 and 2). Most clinics (92%) reported HIV-1/HIV-2 Ag/Ab availability, but fewer than two in five clinics reported p24 (37%) and HIV-1/2 Ab suppl (40%) availability. Viral load availability was reported by half of the clinics (50%), as was CD4 (53%). Genotyping was available at 30% of clinics. Clinics reporting test availability were predominantly from the Asia-Pacific or North American regions, served urban populations, and were in high- or middle-income countries, countries without Global Fund or PEPFAR support, and countries having HIV prevalence ≤1%.

### User fees

Among clinics reporting on-site test availability, charging of user fees was generally low (Figures 1 and 2). The percentage reporting user fees ranged from 7% (HIV-1/2 Ag/Ab, genotyping) to 11% (CD4). Across tests, up to 40% of clinics in lower-middle-income countries and 33% in upper-middle-income countries reported user fees, while up to 25% of clinics in countries receiving Global Fund or PEPFAR support reported fees. Except for genotyping, a higher percentage of clinics reporting user fees were from the Asia-Pacific (15%-22% of clinics), Central Africa (17%-100%), or Southern Africa (25%).

### Sub-group analysis

In sub-group analysis of clinics in low- and lower-middle-income countries, across all tests, clinics in PEPFAR-supported countries reported a lower percentage of test availability and a higher percentage of user fees.

## Discussion

We found limited on-site availability of tests for routine HIV diagnosis and management across HIV clinics globally. Further, over 90% of clinics did not report user fees, although this varied by country income status and source of donor funding for HIV.

There was heterogeneity in on-site test availability. Most clinics reported the availability of HIV-1/HIV-2 Ag/Ab. This may likely reflect widespread use of this test for detecting established HIV.^27^ In contrast, p24, HIV-1/2 Ab suppl, and genotyping were least available. It is possible that these more specialized tests were available off-site only, given their higher complexity, higher costs, and/or limited applicability (eg, HIV-1/2 Ab suppl in low HIV-2 prevalence settings).^28,29^ For p24, this test may have been available via HIV-1/HIV-2 Ag/Ab, given the most recent generation of HIV-1/HIV-2 Ag/Ab detects the HIV-1 p24 antigen.

Limited on-site availability for most routine HIV-related tests suggests that test availability and accessibility may be barriers to diagnosis, treatment, and care. For example, genotyping was unavailable on-site at all clinics in low-income countries. This has implications for treatment management, given the test's role in detecting resistance mutations and selecting antiretroviral regimens, for epidemic control, and for equity across country-income settings.^29^ Off-site test availability may offset the low on-site availability observed and therefore increase access, especially for specialized tests like genotyping or confirmatory serology that may be operationally centralized and for which off-site referrals may be standard routine. This contrasts with tests that require on-site availability (eg, point-of-care HIV testing) to be delivered and for which on-site unavailability implies inaccessibility. Integrating HIV-related services, such as point-of-care viral load testing, can improve on-site availability.^30^ However, establishing such service delivery models is costly, with one study finding national scale-up of point-of-care viral load testing costs $50 million annually.^31^

Notably, clinics in Global Fund- or PEPFAR-supported countries generally reported lower availability and higher user fees. These findings persisted for clinics with country-level PEPFAR support in low- and lower-middle-income countries. An explanation is that clinics in these countries, particularly those with higher HIV prevalence, rely on user fees in addition to domestic resources and donor support for the HIV response. Importantly, among clinics in low- and lower-middle-income countries in our study, there was no variation in country-level Global Fund support. Further research is warranted to examine test availability and user fees among HIV clinics with country-level Global Fund support compared to those without country-level Global Fund support in low- and lower-middle-income countries.

A higher percentage of clinics in upper-middle-income countries reported user fees, particularly for HIV diagnostic testing. An explanation—given funding gaps and donor funding that has not kept up with inflation^32^—is that these countries have faced challenges transitioning from reliance on external donors to sustaining the HIV response with domestic funds.^33^ User fees could compensate for funding shortages. Better integration of the HIV response into existing health insurance, such as universal healthcare, could reduce the need for user fees.^34,35^

Differences emerged across regions. Clinics in the Asia-Pacific reported user fees for almost all laboratory tests examined. This region has the second largest population with HIV and approximately one-quarter of new infections globally,^36^ with a 50% funding gap for meeting global targets to end the HIV epidemic.^37,38^ Given 82% of total HIV-related financial resources come from domestic funders,^37^ clinics may implement user fees to offset costs. In contrast, most clinics in North America are in the United States, where the federal Ryan White HIV/AIDS Program and coverage through the Affordable Care Act and Medicaid expansion may reduce or eliminate fees for eligible patients. Threats to the ongoing funding of such US programs may decrease coverage and result in introducing user fees to cover program costs.

Our study is among the first to describe the landscape of user fees across a globally diverse cohort of HIV clinics in countries with different health systems, income levels, and sources of HIV financing. While few clinics reported user fees, shortages of HIV financing may play a role. Indeed, a study conducted in Nigeria found 96% of clinics reintroduced user fees in response to reductions in PEPFAR support.^16^ Our study provides context for research suggesting associations between the introduction of user fees and reduced HIV diagnosis,^39,40^ worsened new patient enrollment, and decreased ART prescriptions,^8^ as well as the positive association of free antiretroviral therapy with viral suppression.^41^ This suggests the abrupt withdrawal of PEPFAR funding in 2025 may trigger the reintroduction of user fees globally, worsen HIV-related healthcare utilization, and hinder progress toward ending the HIV epidemic.^8^

Our work has implications beyond HIV. Our findings on the availability of HIV laboratory diagnostics may be comparable to the availability of diagnostics for other conditions. For example, across six common conditions (diabetes, hypertension, HIV, tuberculosis, hepatitis B, and syphilis (pregnant women only)), approximately half of the global population is estimated to have low access to diagnostic tests,^42,43^ and limited availability of cancer-related diagnostic tests hinders timely diagnosis in low-and middle-income countries.^44^ This impacts cascades of care, with limited laboratory test availability contributing to increased morbidity and mortality. While charging of user fees was generally low in our sample, out-of-pocket spending, which includes user fees, represents a high percentage of health expenditures in many low- and middle-income countries and points to the importance of user fees as a source of local health financing in these settings.^45,46^ With recent precipitous decreases in donor funding across health conditions,^47^ low- and middle-income countries must move toward self-sustainability. Given that user fees may be inefficient for increasing overall health sector financing,^48^ countries should prioritize alternative financing mechanisms (eg, prepayment schemes such as taxation) and health sector fund allocation to raise healthcare resources.^49^

### Limitations

First, in restricting our definition of availability to on-site only vs on-site and off-site, we may have underestimated the availability of tests, particularly those that may be centralized or higher-complexity (eg, genotyping). Second, we did not collect information on the amount of user fees charged and cannot assess associated financial barriers for HIV diagnosis, treatment, and care. We also did not collect information on the existence of means-tested waivers or related exemptions, which did not allow us to capture real-world heterogeneity in user fees. Third, our categorizations of PEPFAR and Global Fund country support may not reflect support at the clinic level, as PEPFAR and the Global Fund do not support all clinics within a country. Fourth, we relied on site-level self-report, which may be subject to social desirability bias (ie, respondents may not wish to report user fees) and other information biases (eg, respondents may not have fully identified all types of user fees occurring for patients). Finally, most participating clinics served urban populations, and urban clinics may be better resourced than peripheral rural clinics. Accordingly, our findings may overestimate test availability.

## Conclusions

While the availability of critical HIV-related laboratory tests was generally limited among a diverse global cohort of HIV clinics, most clinics did not report user fees in 2019. Continued monitoring of user fees for essential HIV-related laboratory tests may provide insights about how health systems are responding to steep reductions in global funding for the HIV response.

## Supplementary Material

qxag151_Supplementary_Data

## Data Availability

The data that support the findings of this study are available from the corresponding author upon reasonable request. A request to access the survey data from the IeDEA consortium for research purposes may be made through submission of a formal concept proposal, which is available at https://www.iedea.org/.

## Publication history

A previous version of this manuscript was posted to medRxiv: doi: https://doi.org/10.64898/2025.12.05.25341715.
